# Novel Mechanistic Insights into Primary Biliary Cholangitis: From Pathogenesis to Mesenchymal Stem Cell-Mediated Repair

**DOI:** 10.3390/biomedicines14051101

**Published:** 2026-05-13

**Authors:** Zhenxia Huang, Meiling Zhang, Xiaoyue Zhang, Yao Ge, Cuifang He, Junfeng Li

**Affiliations:** 1 The First Clinical Medical College, Lanzhou University, Lanzhou 730000, China; 2 Department of Hepatology, The First Hospital of Lanzhou University, Lanzhou 730000, China; 3 Institute of Infectious Diseases, The First Hospital of Lanzhou University, Lanzhou 730000, China

**Keywords:** primary biliary cholangitis, mesenchymal stem cells, exosomes, extracellular vesicles, therapy

## Abstract

Primary biliary cholangitis (PBC) is an autoimmune-mediated cholestatic liver disease characterized by the progressive destruction of intrahepatic bile ducts, which ultimately leads to hepatic fibrosis and cirrhosis. The current first-line therapy, ursodeoxycholic acid, is associated with a high rate of non-response. Moreover, second-line treatments are constrained by variable efficacy and safety concerns. Mesenchymal stem cells (MSCs), owing to their potent immunomodulatory and tissue-repairing capabilities, represent a promising new therapeutic strategy for PBC patients with poor response to conventional therapies. This review systematically outlines the pathogenesis of PBC, focusing on factors including genetics, environment, and immune dysregulation. Furthermore, it examines recent evidence on the mechanisms by which MSCs and their derivatives, such as exosomes, may intervene in PBC progression through immunomodulation, anti-fibrotic effects, and potential hepatic differentiation. This paper also reviews the current status and challenges of the clinical translation of MSCs therapy, and proposes that engineered modification and standardized preparation are the key directions to promote its application. In conclusion, this review provides a theoretical foundation and future directions for deepening the understanding of PBC pathogenesis and developing novel MSC-based therapeutic strategies.

## 1. Introduction

Primary biliary cholangitis (PBC) is an autoimmune liver disease characterized by high-titer positive anti-mitochondrial antibodies (AMA) and specific antinuclear antibodies, intrahepatic bile duct injury, cholestasis, progressive liver fibrosis, and ultimately decompensated cirrhosis [1]. Globally, PBC is not a rare disease, with an estimated annual incidence of 1.76 per 100,000 and a prevalence of 14.6 per 100,000. The disease exhibits a marked gender bias, predominantly affecting middle-aged women [1]. The current first-line treatment for PBC, ursodeoxycholic acid (UDCA), has a non-response rate as high as 40%. Second-line therapies such as obeticholic acid (OCA) and fibrates face limitations including worsening pruritus, risks of hepatic decompensation, and variable individual efficacy. The recently approved peroxisome proliferator-activated receptor (PPAR) agonists elafibranor and seladelpar offer additional options, yet their use is constrained by dose-dependent safety concerns, contraindications in specific populations, and reliance on surrogate biochemical endpoints rather than clinical outcomes [2,3]. End-stage patients rely on liver transplantation with high recurrence rates, necessitating the development of safe and effective new therapies [4].

Mesenchymal stem cells (MSCs) are a type of adult stem cell with self-renewal and multipotent differentiation capabilities, exhibiting potent immunomodulatory and tissue repair functions [5]. Initially recognized for their ability to differentiate into osteogenic, chondrogenic, and adipogenic lineages, subsequent research revealed that their therapeutic benefits rely more on paracrine activity than direct replacement of damaged cells [6]. MSCs secrete diverse bioactive molecules including growth factors, cytokines, chemokines, and extracellular vesicles (EVs). Through mechanisms such as modulating macrophage polarization, suppressing effector T cell and natural killer cell function, and promoting regulatory T cell expansion, they reshape the local immune microenvironment and mitigate tissue injury. Based on these properties, MSCs have been explored for treating refractory autoimmune diseases such as graft-versus-host disease, Crohn’s disease, and systemic lupus erythematosus, yielding encouraging clinical responses in some studies [7].

**Figure 1:**
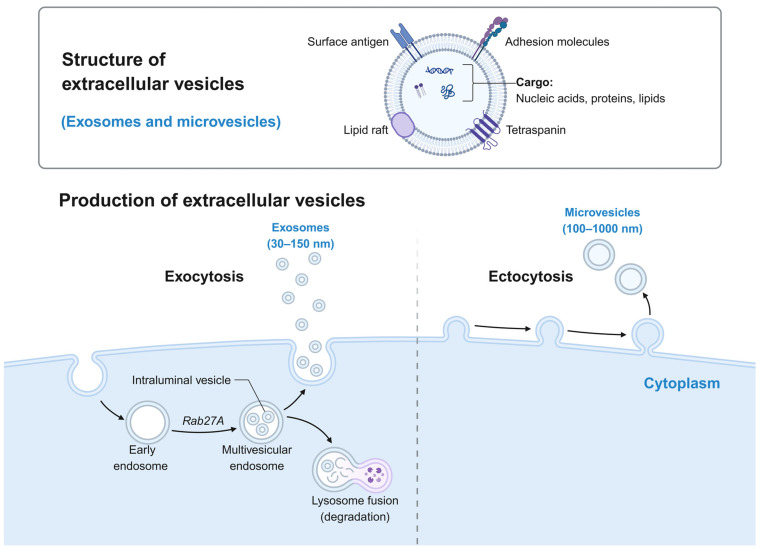
Structure and Production of Extracellular Vesicles. (1) Structure: Extracellular vesicles possess surface antigens, adhesion molecules, tetraspanins, and lipid rafts on their membranes, encapsulating active substances such as nucleic acids, proteins, and lipids; (2) Production: Exosomes (30–150 nm) originate from early endosomes, invaginate to form intraluminal vesicles, and further develop into multivesicular endosomes. Regulated by proteins such as Rab27A, they are released extracellularly via exocytosis. Some multivesicular endosomes may fuse with lysosomes for degradation. Microvesicles (100–1000 nm): Released directly from the cell membrane via ectocytosis. Created in BioRender. Zhenxia Huang (2026) https://app.biorender.com/illustrations/698d8782e455b6a0edf9078b?slideId=72eab32e-848f-4790-b611-df90b94dc57d (accessed on 27 March 2026).

Mesenchymal stem cell-derived extracellular vesicles are the core effector components of paracrine activity. EVs can be classified into exosomes, microvesicles, and apoptotic bodies based on their biogenesis pathways and size, with exosomes being the most extensively studied subgroup. Exosomes are nanoscale membrane vesicles secreted by MSCs, carrying various bioactive molecules including proteins, lipids, and nucleic acids from the parent cells (Figure 1). These vesicles transfer biological information to target cells, thereby exerting functions in immune regulation, anti-apoptosis, and anti-fibrosis. Compared to live cell transplantation, exosomes offer advantages including lower immunogenicity, no tumorigenic risk, and greater physicochemical stability [8]. These characteristics position exosomes as a key representative of “cell-free” regenerative medicine formulations, with their therapeutic potential in autoimmune liver diseases undergoing systematic evaluation.

In this review, we systematically summarize the latest advances in PBC pathogenesis research. Furthermore, we explore the specific molecular mechanisms by which MSCs and their derivatives exert therapeutic effects in PBC. Finally, we evaluate the clinical translation potential of MSC-based therapies for PBC, aiming to provide a theoretical foundation and future directions for novel treatment development.

## 2. Evolving Paradigms in the Pathogenesis of Primary Biliary Cholangitis

**Figure 2:**
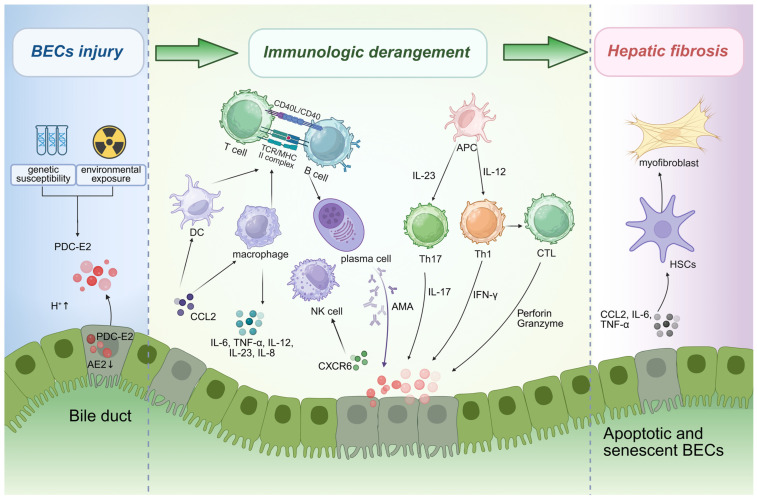
**Evolving Paradigms in the Pathogenesis of Primary Biliary Cholangitis.** The interaction between genetic and environmental factors leads to abnormal exposure of PDC-E2. PDC-E2 is presented by APCs, activating multiple immune cells including Th1, Th17, CTLs, and AMA-producing B cells/plasma cells. Concurrently, innate immune cells exacerbate the inflammatory cycle by releasing pro-inflammatory factors. This self-perpetuating immune attack ultimately activates HSCs, resulting in myofibroblast infiltration and hepatic fibrosis. BECs, Biliary epithelial cells; PDC-E2, Mitochondrial pyruvate dehydrogenase complex E2 subunit; AE2, Anion exchanger 2; DC, Dendritic cell; AMA, Anti-mitochondrial antibodies; NK cell, natural killer cell; APC, Antigen-presenting cell; Th17, Th17 helper T cell; Th1, Th1 helper T cell; CTL, Cytotoxic T lymphocyte; HSCs, Hepatic stellate cells. Created in BioRender. Zhenxia Huang (2026) https://app.biorender.com/illustrations/68f51b596731313bc0c58aef?slideId=6d6bb7a2-2d79-4e57-acd9-06647fff6ac0 (accessed on 27 March 2026).

The core pathogenesis of PBC involves an autoimmune attack by the immune system against bile duct epithelial cells (BECs) in genetically susceptible individuals triggered by environmental factors. This process involves disorders of both innate and adaptive immune systems, leading to progressive damage to the bile ducts (Figure 2).

### 2.1. Genetic Susceptibility and Environmental Exposure

The pathogenesis of PBC involves the combined effects of genetic susceptibility, environmental exposure, and epigenetic regulation. Genome-wide association studies and meta-analyses have identified multiple genetic loci associated with PBC susceptibility, including both HLA and non-HLA regions. HLA loci such as HLA-DQA1*04:01 are susceptibility alleles in European populations, while HLA-DRB1*08:03 is associated with a higher risk in Asian populations [9]. Risk loci in non-HLA regions encompass multiple immune-related genes, including STAT4, IL12A, IL21, CCR6, and CTLA4, which participate in immune cell activation and signal transduction. These genes contribute to pathogenesis by influencing T cell activation, cytokine signaling pathways (e.g., JAK-STAT, IL-12), and immune regulation [10,11]. Notably, certain risk loci exhibit population specificity. For instance, the PTPN2 gene identified in Japanese populations harbors functional variants that disrupt the negative feedback regulation of IFN-γ signaling. This finding deepens our understanding of PBC heterogeneity [12].

Environmental exposure plays a role in triggering and advancing PBC in genetically susceptible individuals. Poor living conditions in early life and prolonged exposure to chemicals (such as hair dyes) are associated with disease onset [13]. Mechanistically, chemical pollutants such as 2,3,7,8-tetrachlorodibenzo-p-dioxin exacerbate autoimmune responses by activating the aryl hydrocarbon receptor pathway, thereby promoting Th1 and Th17 cell differentiation induced by dendritic cells [14]. Notably, certain xenobiotics such as M8OI and 2-octanoic acid disrupt immune tolerance by molecular mimicry. They modify the E2 subunit of the mitochondrial pyruvate dehydrogenase complex (PDC-E2), thereby inducing specific autoimmune responses in genetically susceptible individuals [15,16].

Epigenetic regulation has been demonstrated to bridge genetic susceptibility and environmental exposure [17]. Key mechanisms include DNA hypomethylation at immune gene promoters, histone acetylation-regulated expression of inflammatory mediators, non-coding RNA-mediated cholangiopathy, and X-inactivation escape-associated female predisposition. Genetic, environmental, and epigenetic factors interact to place the immune system in a state of susceptibility. This sets the stage for subsequent activation of innate and adaptive immune responses, which in turn trigger bile duct injury.

### 2.2. Immune Dysregulation

#### 2.2.1. Self-Antigen Presentation

As an autoantigen, PDC-E2 undergoes abnormal exposure and modification, serving as the initial step in triggering autoimmune responses. Damage to the bicarbonate barrier results from downregulation of the primary Cl^−^/HCO_3_^−^ anion exchanger protein 2 (AE2) on the surface of BECs. This damage increases extracellular acidity and heightens the sensitivity of BECs to harmful bile acids. Consequently, cell death is induced, leading to the release of modified PDC-E2 and triggering autoimmune responses [18,19]. In PBC, AE2 downregulation is partially mediated by microRNA-506 (miR-506), which is upregulated in PBC bile duct cells. miR-506 binds to the 3′ untranslated region (3′UTR) of AE2 mRNA, inhibiting its protein translation, resulting in reduced AE2 activity and impaired bile secretion function [20]. Furthermore, miR-506 overexpression activates bicarbonate-responsive soluble adenylate cyclase, sensitizing BECs to bile salt-induced apoptosis, which further exacerbates PDC-E2 release [21]. In PBC, defective glutathione-mediated neutralization of PDC-E2 immunogenicity leads to its exposure on the surface of BECs. PDC-E2 is recognized as an exogenous antigen by autoreactive B cells, triggering AMA production. This recognition stimulates T cell subsets, resulting in immune responses and specific AMA formation [22]. In summary, aberrant exposure and modification of PDC-E2, coupled with impaired bicarbonate secretion by BECs, initiate a cascade of autoantigen presentation that activates both humoral and cellular immune responses, setting the stage for progressive biliary injury—highlighting the BEC as both the target and an active participant in disease initiation.

#### 2.2.2. Congenital Immune Disorders

The abnormal activation of the innate immune system is a key driver of progressive bile duct injury in PBC. BECs recruit monocytes into the liver by releasing chemokines such as CCL2 and CX3CL1 [23]. Upon Toll-like receptor (TLR) stimulation, these monocytes release abundant proinflammatory cytokines, including IL-1β, IL-6, IL-18, IL-12, and TNF-α [24]. Among these, non-classical monocytes are a primary source of IL-8, which activates macrophages—macrophages themselves being another source of IL-8 [25]. Macrophage recognition of the PDC-E2-AMA complex induces a shift toward the proinflammatory M1 phenotype, potentially involving the following processes [26]: (1) Release of high levels of IL-12 to promote Th1 differentiation; (2) Upregulation of TNF-induced apoptosis ligands, directly inducing cell death; (3) Secreting additional proinflammatory mediators. Notably, monocyte-derived macrophages (MoMF) are a major source of IL-23. This cytokine drives IL-17A production, thereby amplifying the Th17 response and exacerbating pericholangitis [27].

Natural killer (NK) cells, as a major component of liver-resident lymphocytes, accumulate significantly in the portal vein region of PBC patients. The liver homing of NK cells is driven by CXCR6, a receptor upregulated upon IL-12 stimulation [28]. Recent studies have also identified an inflammatory-associated NK (irNK) cell subset (CD49a^+^ CXCR6^+^) that accumulates in both PBC mouse models and patient livers. This subset promotes CD4^+^ T cell proliferation by secreting TNF-α, thereby amplifying the immune response [29].

Dendritic cells (DCs) also play a critical role in PBC. Type 2 conventional dendritic cells (cDC2) selectively expand in the liver and acquire proinflammatory monocyte-like characteristics, highly expressing CCL2 and CXCL2 to chemotax infiltrating macrophages and neutrophils. Simultaneously, cDC2 support and sustain Th17 responses by secreting IL-12 and IL-23. This synergizes with CCL20 released by cholangiocytes to promote the sustained recruitment of immature DCs, reinforcing the inflammatory cycle [30]. Conversely, although the cDC1 subset is numerically smaller, its cross-presentation capacity and IL-12 secretion are critical for CD8^+^ T cell activation and portal fibrosis. Its absence significantly attenuates disease severity [31].

Mucosa-associated innate-like T (MAIT) cells constitute a T cell subset resembling innate immunity, exhibiting increased hepatic infiltration and reduced peripheral blood levels in PBC patients [32]. This liver-specific accumulation is mediated by the CXCL12-CXCR4 chemokine axis. MAIT cells in PBC patients highly express CXCR4, while CXCL12 levels are upregulated in diseased livers, jointly driving MAIT cell liver homing [33]. MAIT cell function in PBC pathogenesis is regulated by multiple signaling pathways. On one hand, bile acids induce hepatocytes to produce IL-7, which activates the STAT5 phosphorylation pathway within MAIT cells. This promotes granzyme B and inflammatory cytokine production, thereby bridging cholangiocyte injury and innate immune activation [32]. On the other hand, elevated IL-18 in PBC patient plasma further stimulates MAIT cells to produce IFN-γ via its receptor (IL-18Rα), exacerbating portal vein inflammation [33].

Additionally, neutrophils exacerbate pericholangial oxidative damage and pyroptosis by producing reactive oxygen species (ROS) and neutrophil extracellular traps (NETs), with NET components serving as potential therapeutic targets [34,35]. Under steady-state conditions, neutrophils clear apoptotic hepatocytes via “perforocytosis”. Impairment of this clearance function may contribute to autoantibody production and disease progression [36].

In summary, innate immune cells in PBC form a self-perpetuating inflammatory network through complex chemokine, cytokine, and cell–cell interactions, leading to progressive bile duct injury through oxidative stress and apoptosis. Dual-targeted therapies addressing innate immune cells and their crosstalk with adaptive immunity may offer novel strategies for controlling proinflammatory responses in cholestatic liver diseases.

#### 2.2.3. Adaptive Immune Disorders

Concurrent with the activation of the innate immune system, adaptive immune responses—particularly the pathological expansion and persistence of autoreactive T cells—contribute to the specificity and persistence of biliary tract damage in PBC. Current understanding suggests that in the early stages of PBC, the ultimate damage to bile duct epithelium is driven by Th1 immune responses, while in later stages, the damage is sustained by Th17 cells. IL-12 and IL-23 are mainly produced by antigen-presenting cells and are responsible for promoting Th1 and Th17 immune responses respectively [37]. Concurrently, cholangiocytes also secrete IL-6, IL-1β, and IL-23 to recruit Th17 cells around bile ducts, exacerbating local inflammation [38]. The portal zone of PBC patients harbors abundant terminally differentiated cytotoxic CD8^+^ T cells, among which a subset of CD103^+^ tissue-resident memory (TRM) T cells specifically targeting PDC-E2 has been identified as the primary autoreactive and cytotoxic cells in PBC liver. TRM cells persistently reside in tissues with limited migration capacity, suggesting a strong association with PBC chronicity and organ-specific damage. Huang et al. demonstrated that the long-term residency of TRM cells is regulated by N-acetyl-D-glucosamine 4-deoxyribosyl transferase 1, which promotes chronic inflammation by maintaining oxidative stress tolerance [39]. Recent studies identified a CD8^+^ T cell subset co-expressing TRM markers (CD69^+^ CD103^+^) and E-cadherin. This subset abnormally overexpresses E-cadherin upon TCR activation. E-cadherin^+^ CD8^+^ T cells form β-catenin-mediated interactions with BECs, driving CD8^+^ T cell invasion into BECs and exacerbating biliary duct injury [40]. B cells target PDC-E2 antigens by producing autoantibodies (AMA), whose immune complexes cross-presented by dendritic cells amplify autoreactive T cell activation. Furthermore, plasma cells play a central pathogenic role in PBC, as their depletion reduces portal vein inflammation and autoantibody levels, whereas B cell depletion lacks this effect [41].

In conclusion, the immune dysregulation in PBC involves a coordinated interplay between innate and adaptive immunity. Aberrant presentation of the self-antigen PDC-E2 triggers an initial autoimmune response, which is subsequently amplified by innate immune cells including monocytes, macrophages, NK cells, and dendritic cells. These cells release pro-inflammatory cytokines that promote the differentiation and persistence of autoreactive T cells and B cells. Once established, the adaptive immune response becomes self-sustaining through a distinct mechanism: autoreactive CD4^+^ and CD8^+^ T cells drive biliary epithelial damage, while B cells and plasma cells perpetuate the response via continuous autoantibody production. This interdependence suggests that effective immunotherapy may need to target both T and B cell compartments simultaneously.

### 2.3. Apoptosis and Senescence of BECs

BECs are not only targets of immune-mediated injury but also participate in disease progression through apoptosis and senescence. Although these two mechanisms are mutually exclusive, they jointly drive the process of bile duct injury. Studies indicate that BECs in PBC patients overexpress TNFα and Fas/FasL, triggering apoptosis via the extrinsic death receptor pathway [42]. Concurrently, mitochondrial stress or the unfolded protein response can activate the intrinsic apoptotic pathway. Both extrinsic and intrinsic pathways ultimately converge on caspase activation to execute cell death [43]. Furthermore, gut microbiota dysbiosis promotes BEC apoptosis via the TLR2 signaling pathway and induces CXCL10 secretion, which further recruits CD8^+^ T cells to exacerbate biliary tract injury [44].

Under conditions of persistent injury, the primary response of BECs may shift from apoptosis toward cellular senescence. Senescent BECs exhibit permanent cell cycle arrest (at G_1_ or G_2_ phase) and resistance to apoptosis, which correlates with overexpression of anti-apoptotic mediators such as Bcl-2 and Bcl-Xl [45]. Senescent cells enter a state of excessive secretion, known as the senescence-associated secretory phenotype (SASP), releasing multiple pro-inflammatory factors (e.g., IL-6, TNF-α), chemokines, and pro-fibrotic factors (e.g., TGF-β, MCP-1), thereby exacerbating biliary injury and fibrosis [46]. Dysregulation of the Hippo-YAP pathway also contributes to bile duct epithelial senescence, with reduced YAP1 expression enhancing cellular senescence and inhibiting proliferation [47].

Studies indicate that markers of cholangiocyte senescence (p^16INK4a^, p^21WAF1/Cip1^) show a more pronounced increase in advanced PBC [48]. Notably, increased p^16INK4a^ expression is independently associated with poor response to UDCA treatment, suggesting that senescent cell accumulation may impair drug efficacy [49]. Studies in Mdr2^−/−^ mice further confirm that eliminating senescent bile duct cells through genetic or pharmacological interventions significantly ameliorates biliary tract lesions. Results demonstrate that these interventions markedly reduce expression of senescence markers p16 and p21 in the liver, suppress release of key inflammatory mediators (TNF-α, IL-1β, MCP-1), and significantly attenuate hepatic fibrosis [50]. Furthermore, estrogen receptor α (ERα)-mediated mitochondrial damage may also promote BEC senescence and apoptosis, further elucidating the complexity of PBC [51]. In summary, targeting senescent BECs has emerged as a potential therapeutic strategy aimed at blocking disease progression and promoting tissue repair.

### 2.4. Inflammation and Fibrosis

The pathogenesis of PBC involves the interaction between cholangiocyte-centered inflammatory responses and fibrotic processes. Following infection or tissue injury, cholangiocytes become activated and release pro-inflammatory cholangiokine factors such as CCL2, IL-6, and TNF-α. These factors initiate and sustain a local inflammatory state, subsequently activating hepatic stellate cells (HSCs) and portal fibroblast cells. This leads to excessive deposition of the extracellular matrix (ECM), ultimately resulting in liver fibrosis and even cirrhosis.

Persistent bile duct injury activates multiple inflammatory signaling pathways. Sustained activation of the NF-κB pathway is a key component, upregulating proinflammatory factors and adhesion molecule expression while interacting with Wnt/β-catenin signaling to regulate inflammatory and fibrotic progression during ductal responses [52]. Toll-like receptors (TLRs), particularly TLR4, respond to microbial stimuli and lipopolysaccharides, thereby activating N-Ras to produce proinflammatory cytokines. TLR4 also activates NF-κB via myeloid differentiation protein 88 and IL-1 receptor-associated kinase, inducing bile duct cells to secrete more inflammatory mediators while impairing bile duct bicarbonate secretion function, amplifying bile acid cytotoxicity [53]. Bile acid dysregulation itself constitutes a significant proinflammatory factor. Downregulation of the bile acid membrane receptor TGR5 or dysfunction of the nuclear receptor FXR disrupts bile acid metabolism, thereby promoting inflammation and reactive bile duct hyperplasia [54].

The inflammatory response subsequently promotes the progression of fibrosis. TGF-β signaling serves as a core driver of fibrosis, facilitating the transformation of HSCs into myofibroblasts and stimulating the extensive synthesis and deposition of ECM. The Wnt pathway enhances bile duct cell proliferation and ductal reactivity, forming crosstalk with pathways such as TGF-β and Hedgehog to collectively amplify the fibrotic response [55]. The inflammatory and fibrotic processes in PBC form a mutually reinforcing vicious cycle. Interventions targeting key signaling nodes—such as TGF-β, TLR, NF-κB, or bile acid receptors—may offer potential therapeutic avenues for PBC.

In brief, the pathogenesis of primary biliary cholangitis arises from a complex interplay among genetic susceptibility, environmental triggers, and multifaceted immune dysregulation, ultimately leading to injury of the biliary epithelial cells. Innate immune cells initiate and amplify the inflammatory response, adaptive immunity perpetuates chronic autoimmunity, and damage to the biliary epithelial cells drives a continuous cycle of injury and repair. This integrated framework highlights the multiple points at which therapeutic interventions—including mesenchymal stem cell-based strategies—may interrupt disease progression. In line with this framework, the immunomodulatory properties of MSCs directly counter the innate and adaptive immune imbalances central to PBC, while their anti-fibrotic and pro-regenerative capacities address the subsequent biliary epithelial injury and fibrotic remodeling.

## 3. Mechanisms of MSCs and Their Derivatives in Treating PBC

**Figure 3:**
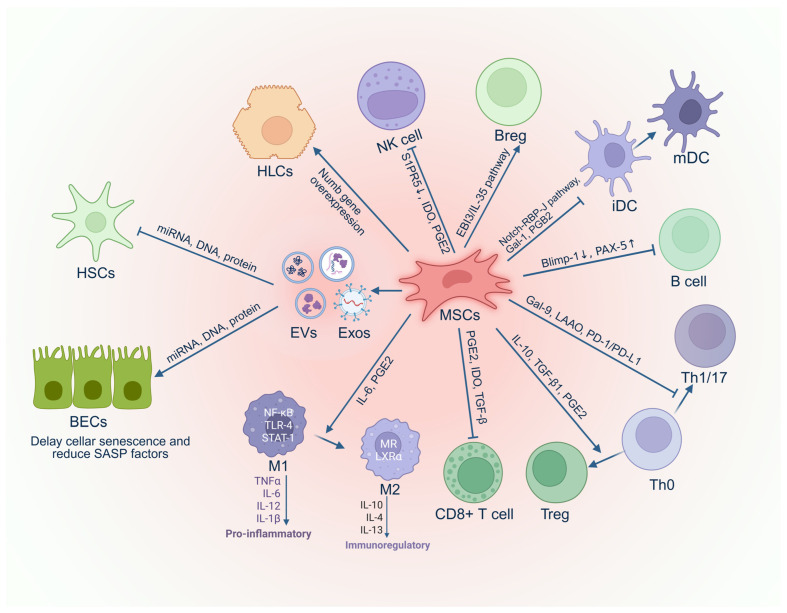
**Mechanism of MSCs and Their Derivatives in Treating PBC.** MSCs, Mesenchymal stem cells; Exos, Exosomes; EVs, Extracellular vesicles; HSCs, Hepatic stellate cells; BECs, Biliary epithelial cells; SASP, Senescence-associated secretory phenotype; M1, M1-type macrophages; M2, M2-type macrophages; Treg, Regulatory T cell; Th0, Naive Th cells; Th1/17, Th1 and Th17 cells; iDC, Immature dendritic cell; mDC, Mature dendritic cell; Breg, Regulatory B cell; NK cell, natural killer cell; HLCs, Hepatocyte-like cells. Created in BioRender. Zhenxia Huang (2026) https://app.biorender.com/illustrations/68ebdccb34d69b0f3ed80a04?slideId=295f9c5b-c538-48ad-8eac-49c690f5b6cb (accessed on 27 March 2026).

MSCs are a type of adult stem cell with multipotent differentiation potential and immunomodulatory functions. They are widely distributed in tissues such as bone marrow, adipose tissue, and the umbilical cord. The traditional paradigm held that MSC therapy primarily relied on direct differentiation into tissue cells to replace damaged ones; however, accumulating evidence now indicates that therapeutic benefits are exerted chiefly through the secretion of diverse bioactive factors. These factors modulate the local microenvironment, suppress inflammatory responses, and promote tissue repair [6] (Figure 3). In recent years, MSC therapeutic strategies have been shifting from cell transplantation toward “cell-free” approaches. Among these, EVs secreted by MSCs—and their primary subtype, exosomes (Exos)—serve as crucial mediators of intercellular communication. These vesicles can carry and transport functional cargo such as proteins, lipids, and nucleic acids (e.g., mRNA, miRNA, DNA) to recipient cells, thereby altering their biological behavior.

### 3.1. Immunomodulation

Given the central role of immune dysregulation in PBC pathogenesis, MSCs offer a mechanistic rationale for therapeutic intervention by targeting these abnormalities. As detailed in the preceding sections, PBC is characterized by an imbalance between pro-inflammatory effector T cells (Th1 and Th17) and regulatory T cells (Tregs), along with aberrant B cell activation and autoantibody production. MSCs address these pathological features through multiple coordinated mechanisms that restore immune homeostasis.

#### 3.1.1. T Cells

**Figure 4:**
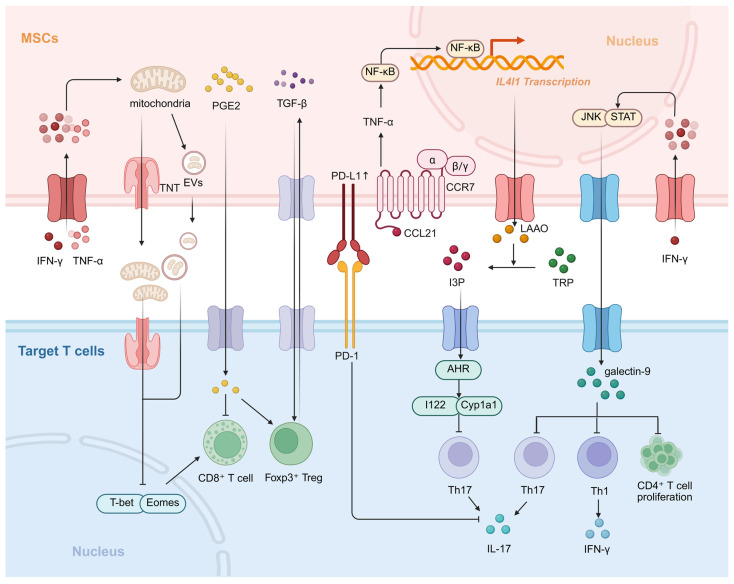
**Molecular mechanisms by which MSCs regulate T cell responses.** In primary biliary cholangitis, autoreactive T cells drive inflammation and injury. MSCs act through the following coordinated molecular pathways: (1) Mitochondrial transfer via TNTs or EVs, delivering functional mitochondria to CD8^+^ T cells, downregulating key transcription factors, and suppressing their cytotoxic function; (2) Secretion of soluble factors promoting the differentiation of naive CD4^+^ T cells into Foxp3^+^ Tregs; (3) High expression of PD-L1, binding to the PD-1 receptor on T cells; (4) Secretion of LAAO, catalyzing TRP metabolism and suppressing Th17 responses in local lymph nodes via its metabolites; (5) Secretion of galectin-9, inhibiting the differentiation of CD4^+^ T cells into Th1/Th17 subsets. MSCs, Mesenchymal stem cells; TNTs, Tunneling nanotubes; EVs, Extracellular vesicles; PGE_2_, prostaglandin E_2_; TGF-β, Transforming growth factor-β; Treg, Regulatory T cell; PD-L1, Programmed death-ligand 1; PD-1, Programmed death-1; LAAO, L-amino-acid oxidase; TRP, Tryptophan; I3P, Indole-3-pyruvic acid; AHR, Aryl hydrocarbon receptor. Created in BioRender. Zhenxia Huang (2026) https://app.biorender.com/illustrations/6933b06f87eb971fb75ed4e5?slideId=a4796f57-5bf9-41cc-9134-da3f2334c68b (accessed on 28 March 2026).

During the progression of PBC, subsets of autoreactive T cells undergo dynamic changes, with each subset playing distinct pathogenic roles at different stages of the disease. In the early stages, Th1-type immune responses predominate; as the disease progresses, Th17-type immune responses gradually become dominant [37]. Meanwhile, CD8^+^ T cells exert a cytotoxic effect on BECs. MSCs precisely regulate pathogenic T cells of different subtypes at these various stages while promoting the generation of Tregs, thereby restoring immune balance (Figure 4).

In the early stages of PBC, Th1 cells and the IFN-γ they secrete are the primary pathogenic factors. MSCs inhibit the Th1 differentiation of CD4^+^ T cells by secreting galectin-9 (Gal-9) [56]. As the disease progresses to the chronic phase, Th17 cells gradually become dominant, and MSCs suppress their differentiation and function through multiple mechanisms: on the one hand, MSCs upregulate the expression of L-amino acid oxidase (LAAO) via the NF-κB pathway in the inflammatory microenvironment, catalyzing tryptophan metabolism to generate specific products that activate the aromatic hydrocarbon receptor (AHR) pathway, thereby inhibiting Th17 cell differentiation [57]; on the other hand, by highly expressing PD-L1, MSCs bind to PD-1 on the surface of T cells, thereby inhibiting the differentiation and function of Th17 cells and reducing IL-17 production [58]. Furthermore, Gal-9 secreted by MSCs can also suppress Th17 cell generation, achieving dual regulation of both Th1 and Th17 cells [56]. In experimental autoimmune cholangitis and autoimmune hepatitis models, MSC therapy significantly reduced Th1 and Th17 cell proportions while improving hepatic inflammation [56,59]. Earlier studies revealed that MSCs suppress CD8^+^ T cell proliferation and cytokine production by downregulating NKG2D activation receptor expression on their surface and secreting soluble factors such as PGE_2_, IDO, and TGF-β [60]. Recent studies further demonstrated that MSCs transfer mitochondria to CD8^+^ T cells via direct cell-to-cell contact, thereby downregulating key transcription factors T-bet and Eomes to inhibit their proliferation. This pathway synergizes with MSC-secreted PGE_2_ to collectively attenuate the responsiveness of CD8^+^ T cells [61]. The immunomodulatory functions of MSCs do not always act directly on T cells; sometimes they rely on other immune cells as mediators. For example, the inhibitory effect of umbilical cord-derived MSCs (UCMSCs) on T cell proliferation depends on the presence of monocytes. UCMSCs indirectly suppress T cell proliferation by modulating the phenotype of monocytes and inducing them to produce PGE_2_ [62].

While suppressing pathogenic T cells, MSCs promote the establishment of an immune tolerance environment. Co-culturing with MSCs during the early stages of Th1/Th17 cell differentiation promotes the generation of CD4^+^ CD25^+^ Foxp3^+^ Tregs and upregulates the secretion of the anti-inflammatory factor IL-10, thereby establishing the foundation for immune regulation [63]. Furthermore, MSCs promote Treg differentiation by secreting factors such as IL-10, TGF-β1, and prostaglandin E_2_ (PGE_2_). The resulting Tregs are predominantly of an induced phenotype (iTregs), which are known to suppress the proliferation of activated CD4^+^ T cells. Moreover, MSCs can induce apoptosis in activated T cells via the Fas/FasL pathway. The apoptotic T cells subsequently trigger macrophages to secrete TGF-β, which further promotes Treg generation, forming a virtuous cycle from cell clearance to immune tolerance [64]. Moreover, TGF-β derived from Tregs can also enhance the immunoregulatory and tissue repair capabilities of MSCs through feedback mechanisms [65], suggesting that the combined use of Tregs and MSCs may represent a more promising therapeutic strategy.

To further enhance their therapeutic potential, genetically modified MSCs demonstrate superior efficacy. For instance, MSCs overexpressing soluble fibrinogen-like protein 2 (sFgl2) more effectively promote Treg differentiation and suppress Th1/Th17 responses [66]. Concurrently, MSCs co-expressing PD-L1 and intercellular adhesion molecule-1 (ICAM1) (PI-MSCs) not only enhance homing capacity to injured liver tissue but also more effectively upregulate Treg ratios via PD-L1-mediated signaling. PI-MSCs demonstrate superior therapeutic efficacy in autoimmune hepatitis models [67].

#### 3.1.2. B Cells

MSCs directly inhibit B cell proliferation and differentiation through soluble factors. MSCs significantly suppress B cell proliferation and arrest their cell cycle at the G_0_/G_1_ phase [68,69]. MSCs block the terminal differentiation of B cells into plasma cells by downregulating the key plasma cell transcription factor Blimp-1 and upregulating PAX-5, leading to a significant decrease in the proportion of CD138^+^ plasma cells and reduced secretion of IgM, IgG, and IgA [69]. This process involves phosphorylation modifications in the Akt and p38 MAPK signaling pathways, and Transwell experiments confirm its dependence on soluble factors rather than direct cell-to-cell contact. Furthermore, in liver fibrosis models, MSC-derived exosomes have been shown to further inhibit B cell activation by suppressing the MAPK and NF-κB pathways, thereby enhancing the anti-fibrotic effect [70].

MSCs exert their immunomodulatory effects by inducing regulatory B cells (Bregs), thereby modulating the immunosuppressive microenvironment. Adipose mesenchymal stem cells (ADMSCs) promote B cell differentiation toward a CD19^+^ CD24^+^ CD38^+^ transitional phenotype, a population that highly secretes IL-10 that mitigates B cell-mediated pathological immune responses. These induced Bregs (iBregs) effectively suppress T cell proliferation in a dose-dependent manner. Transcriptomic analysis further revealed Breg heterogeneity, identifying a distinct IL-10^−^ iBreg subset enriched for extracellular matrix genes and TGF-β signaling pathways. The immunoregulatory function of this subset is independent of IL-10 but dependent on TGF-β [71]. Notably, the regulatory effects of MSCs exhibit environment dependence [72]: under inflammatory quiescence, MSCs promote Breg generation and IL-10 secretion without inhibiting B cell proliferation. Conversely, during active inflammation, MSCs shift to suppressing B cell proliferation and antibody production while losing their capacity to induce Bregs. However, the mechanisms by which MSCs regulate B cells show source- and T cell-dependent variations. ADMSCs directly inhibit plasmacytoma formation and induce Bregs, with this effect independent of T helper cells [73]. Conversely, the suppression of B cell proliferation and antibody production by bone marrow-derived mesenchymal stem cells (BMSCs) strictly depends on the presence of T cells and direct contact between MSCs and T cells, suggesting that the mechanism of action of MSCs may vary across different microenvironments [74].

Further studies reveal that MSCs regulate B cell fate through metabolic and intercellular communication pathways. For instance, MSCs can mediate the intercellular transfer of functional mitochondria to immune cells. Although CD19^+^ B cells receive fewer mitochondria, those that do exhibit higher survival rates and a low activation state. This process correlates with ROS levels and glycolytic activity [75].

#### 3.1.3. Macrophages

Macrophages are key cells in the innate immune system, playing crucial roles in inflammatory responses, tissue repair, and fibrosis. By polarizing macrophages toward an anti-inflammatory M2 phenotype, MSCs exert both immunomodulatory and tissue-repairing effects. These MSC-regulated macrophages are termed “MSC-educated macrophages (MEMs)”. MEMs highly express M2-type surface markers such as CD206 and CD163, upregulate the secretion of anti-inflammatory factors like IL-10 and TGF-β, and simultaneously downregulate the expression of pro-inflammatory factors such as IL-12 and TNF-α. Furthermore, MEMs show positive correlations in gene expression profiles with multiple tissue repair-related pathways (e.g., epithelial–mesenchymal transition, collagen formation), suggesting their potential to promote regeneration [76].

Macrophage polarization is regulated by MSCs through the secretion of multiple soluble factors. Among these factors, IL-6 is a key driver of M2b polarization. Preconditioning enhances MSCs’ IL-6 secretion capacity, thereby promoting macrophage conversion to the M2b phenotype and suppressing T cell IFN-γ production [77]. PGE_2_ also serves as a key MSC-mediated macrophage regulatory mediator, activating STAT6 and mTOR signaling pathways via the EP4 receptor to promote M2 polarization [78]. Li et al. revealed the dual regulatory role of BMSCs in alleviating liver fibrosis through macrophage-mediated mechanisms. On one hand, MSCs promote the conversion of Ly6C^i^ to Ly6C^lo^ macrophages by secreting IL-4 and IL-10, thereby alleviating liver fibrosis. On the other hand, following MSC apoptosis in vivo, their apoptotic bodies are phagocytosed by Ly6C^lo^ macrophages. This triggers the PtdSer-MerTK-ERK signaling pathway, which upregulates MMP12 expression and promotes extracellular matrix degradation, thereby alleviating liver fibrosis [79]. These findings systematically reveal the macrophage-mediated therapeutic effects of MSCs in multiple liver disease models, providing cellular and molecular mechanisms for the application of MSCs in immune-mediated liver diseases such as PBC.

#### 3.1.4. NK Cells

NK cells play a pivotal role in innate immunity and inflammatory responses within the liver, while MSCs can influence the progression of immune-mediated liver disease by regulating NK cell migration, activation, and function. MSCs inhibit NK cell cytotoxicity and IFN-γ production by secreting IDO and PGE_2_. This inhibitory effect is consistently observed in non-contact Transwell co-culture, whereas direct contact induces variable immunomodulation depending on the NK cell line: KHYG-1 cells exhibit significantly reduced cytotoxicity, whereas NK-92 cells maintain high killing capacity and can lyse MSCs [80]. Furthermore, MSCs inhibit IL-2-activated NK cell proliferation and downregulate expression of activation receptors including NKp30, NKp44, and NKG2D, without affecting inhibitory receptors. IDO and PGE_2_ exert synergistic inhibitory effects in this process [81]. Moreover, IL-15-expressing mesenchymal stromal cells in bone marrow exhibit functional heterogeneity. Among these, LepR^+^ MSCs and sinusoidal endothelial cells regulate NK cell development, survival, and memory T cell homeostasis at distinct stages, suggesting unique regulatory roles for different MSC subtypes within the immune microenvironment [82]. Researchers have proposed strategies to enhance their therapeutic potential: proinflammatory cytokine-induced MSCs (CK-MSCs) have been shown to significantly amplify their immunosuppressive capacity against NK cells. Even at low MSC:NK ratios, CK-MSCs effectively inhibit NK cell proliferation and NKG2D expression while reducing TNF-α secretion, simultaneously decreasing therapeutic heterogeneity among donors [83].

#### 3.1.5. DCs

DCs, as key antigen-presenting cells, play a central role in immune responses. MSCs influence the course of immune responses through regulation of DC differentiation, maturation, and function. Suppression of DC maturation by MSCs is characterized by downregulation of co-stimulatory molecules and major histocompatibility complex (MHC)-II, accompanied by reduced IL-12 secretion and diminished T cell-stimulating capacity [84]. In addition, MSCs promote the differentiation of DCs into regulatory DCs, a phenotype characterized by high expression of IL-10, TGF-β, and IDO, along with the ability to promote Treg differentiation. These regulatory DCs play a crucial role in alleviating liver injury and regulating immune tolerance [85].

Overall, MSCs orchestrate a broad-spectrum immunomodulatory program that simultaneously targets multiple cellular players in the PBC immune network. They suppress effector T cells, NK cells, and pro-inflammatory M1 macrophages while promoting the expansion of Tregs, regulatory B cells, and anti-inflammatory M2 macrophages. They also inhibit the maturation and antigen-presenting function of dendritic cells. This multi-cell, multi-mechanism regulation enables MSCs to intervene at multiple nodes of the pathogenic immune cascade—from initial antigen presentation to effector cell activation and tissue injury—thereby restoring immune homeostasis in the liver.

### 3.2. Hepatic Differentiation Potential

In mouse models of liver injury, MSCs can differentiate into hepatocyte-like cells (HLCs) in vivo, thereby restoring liver function [86]. However, increasing clinical applications indicate that only a small fraction of MSCs undergo differentiation yet still produce effective therapeutic outcomes. This suggests that MSCs exert their therapeutic effects primarily through the secretion of bioactive factors and immune-modulating actions mediated by interactions with immune cells. Xu et al. discovered that the Numb gene is a key regulator determining the differentiation direction of BMSCs [87]. Overexpression of Numb promotes differentiation into hepatocytes, suppressing bile duct reactivity and fibrosis; conversely, Numb knockdown accelerates differentiation into BECs, exacerbating cholestatic liver fibrosis. This study suggests that modulating key molecules like Numb can guide MSCs toward specific differentiation pathways, thereby enhancing their therapeutic potential for PBC.

**Figure 5:**
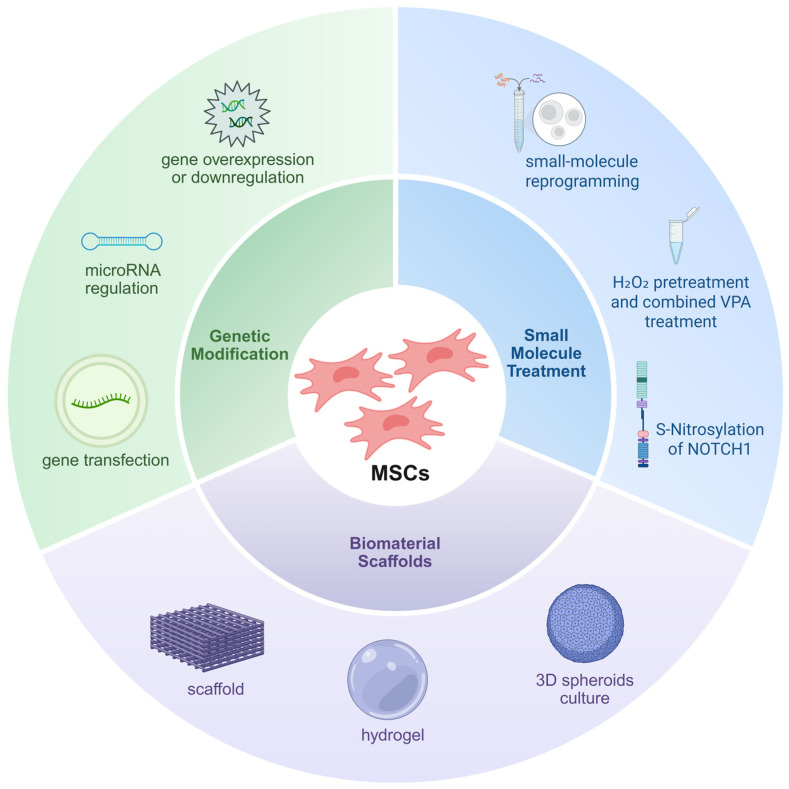
**Intervention strategies to enhance the hepatic differentiation and repair function of MSCs.** This figure summarizes three primary intervention pathways for enhancing the differentiation of MSCs into hepatocyte-like cells and their therapeutic efficacy in PBC through engineered strategies: (1) Genetic modification; (2) Small molecule treatment; (3) Biomaterials scaffold. Created in BioRender. Zhenxia Huang (2026) https://app.biorender.com/illustrations/69258536fcf185161d44c137?slideId=a8be1b85-094d-45d2-ade1-cf205b69794d (accessed on 27 March 2026).

Several preclinical studies have explored strategies to enhance the hepatic differentiation of MSCs (Figure 5). Small molecule-mediated stage-specific reprogramming efficiently differentiates rat BMSCs into functional hepatocyte-like cells within 14 days, significantly improving hepatic function parameters and tissue injury in acute liver injury models [88]. Combined hydrogen peroxide pretreatment with valproic acid synergistically enhances BMSCs’ migration, proliferation, and hepatogenic differentiation capacity [89]. Inhibiting the Notch signaling pathway significantly promotes MSC differentiation into hepatocyte-like cells and increases expression of hepatic functional markers [90]. Furthermore, a cationic thermosensitive polymer scaffold combined with fibroblast growth factor-4/insulin-like growth factor-1 promotes hepatic differentiation of BMSCs and proliferation of senescent cells, effectively restoring liver function in acute liver failure models [91].

In summary, the therapeutic efficacy of MSCs in PBC is determined not only by their traditional capacities for differentiation and immunomodulation but also by microenvironmental signals and intrinsic gene expression regulation. Future MSC therapies based on gene editing or molecular interventions hold promise for delivering more precise and efficient treatment strategies for PBC.

### 3.3. Anti-Fibrotic Effects

The anti-fibrotic effects of MSCs are primarily achieved through mechanisms such as inhibiting HSCs activation, modulating inflammatory responses, promoting liver regeneration, and degrading the extracellular matrix. MSCs can suppress the proliferation of activated HSCs, promote their apoptosis, and reduce the expression of fibrotic proteins. Transcriptomic analysis suggests that inhibition of the AGE-RAGE signaling pathway may be one of the key mechanisms underlying the anti-fibrotic action of MSCs [92]. Furthermore, MSCs can influence autophagy by upregulating REDD1 expression, thereby inhibiting HSCs’ activation. This process involves the PI3K/AKT/mTOR and TGFβ/Smad pathways, enhancing the anti-fibrotic effect [93]. Systematic reviews further support that MSC transplantation effectively reverses liver fibrosis in animal models, with therapeutic efficacy exhibiting a dose-dependent relationship within a certain range, though excessively high doses offer no additional benefit. Among these, BMSCs demonstrate superior anti-fibrotic effects compared to MSCs from other sources, highlighting the importance of cell origin [94].

MSCs pretreated with different factors significantly enhance therapeutic efficacy. Elzainy et al. compared melatonin (MT)-pretreated AD-MSCs and BMSCs, finding both improved liver function (e.g., reduced ALT, AST, AKP, TBIL) and alleviated fibrosis, inflammation, and apoptosis. MT-pretreated AD-MSCs showed optimal effects, restoring serum markers to near-normal levels [95]. Pre-treatment with IFN-α2 activates the IFNAR1-p-STAT1/2 pathway, prompting MSCs to secrete increased levels of CSF-3, IL-8, and CCL20. This recruits neutrophils to the liver, where MMP8-mediated collagen degradation alleviates fibrosis [96].

### 3.4. Effects of MSC Derivatives

The therapeutic effects of MSCs largely stem from their paracrine functions rather than their direct differentiation capacity [97]. This paradigm shift has directed research interest toward the EVs secreted by MSCs. EVs are a heterogeneous population of lipid-bilayer-enclosed vesicles, which include exosomes and microvesicles among their key subtypes. These vesicles serve as natural carriers for bioactive molecules (e.g., proteins, nucleic acids, lipids), mediating intercellular communication and embodying MSCs’ core therapeutic functions. Among them, Exos represent the most extensively studied subtype. This subsection reviews the potential roles and mechanisms of MSC-derived EVs, particularly Exos, in intervening in the core pathological processes of PBC.

MSC-derived products demonstrate potent regulatory capabilities in correcting immune tolerance imbalances in PBC. MSC-Exos suppress B cell proliferation and activation while reducing antibody levels [98]. Data from Chen et al. indicate that MSC-Exos improve fibrotic conditions by inhibiting Th17 differentiation [99]. In a primary sclerosing cholangitis (PSC) model (an immune-mediated cholangitis akin to PBC), MSC-EVs were found to inhibit IL-17A production by targeting NFKBIZ via the miR-7977 they carry, thereby effectively suppressing Th17 differentiation and alleviating periductal fibrosis [100]. This demonstrates promising therapeutic potential for MSC-derived products in liver fibrosis caused by primary biliary cirrhosis or Th17-associated diseases. Furthermore, MSC-EVs metabolically reprogram CD4^+^ T cells by inhibiting glycolysis and enhancing mitochondrial oxidative phosphorylation, a process dependent on EV-mediated mitochondrial protein transfer, thereby mitigating liver injury [101]. MSC-Exos also broadly modulate innate immunity. They promote macrophage polarization toward the M2 phenotype, for instance, by delivering factors such as miR-182 and IL-10 to improve the inflammatory microenvironment. Furthermore, MSC exosomes suppress DC maturation and NK cell cytotoxicity, thereby modulating immune responses [102]. Addressing the characteristic BEC injury in PBC, another study by Chen et al. further proposed that MSC-EVs exert direct protective effects on bile duct cells. In an oxidative stress-induced bile duct-like cell senescence model, Exos derived from human placental MSCs (hPMSCs) effectively delayed the senescence process and reduced the expression of SASP factors such as IL-6 and CCL2, demonstrating potential bile duct repair functions [103].

MSC-EVs target activated HSCs through multiple mechanisms, reducing abnormal extracellular matrix deposition and exerting potent anti-fibrotic effects. One core mechanism involves regulating key signaling pathways by delivering specific microRNAs. For instance, they alleviate liver fibrosis by suppressing LOXL2 expression through exosome-mediated downregulation of YAP via miR-27b-3p [104]. Furthermore, recent studies reveal that MSC-Exos can induce ferroptosis in HSCs, thereby specifically eliminating activated HSCs. Multiple MSC-derived exosomes target SLC7A11 by delivering miR-26a, miR-144-3p, and others, disrupting intracellular redox balance to trigger ferroptosis and suppress HSC activation [105,106]. Similarly, human umbilical cord MSC (hUC-MSC)-derived exosome miR-499a-5p indirectly promotes HSCs ferroptosis by targeting the transcription factor ETS1, thereby inhibiting its downstream ferroptosis-resistant protein GPX4 [107].

**Figure 6:**
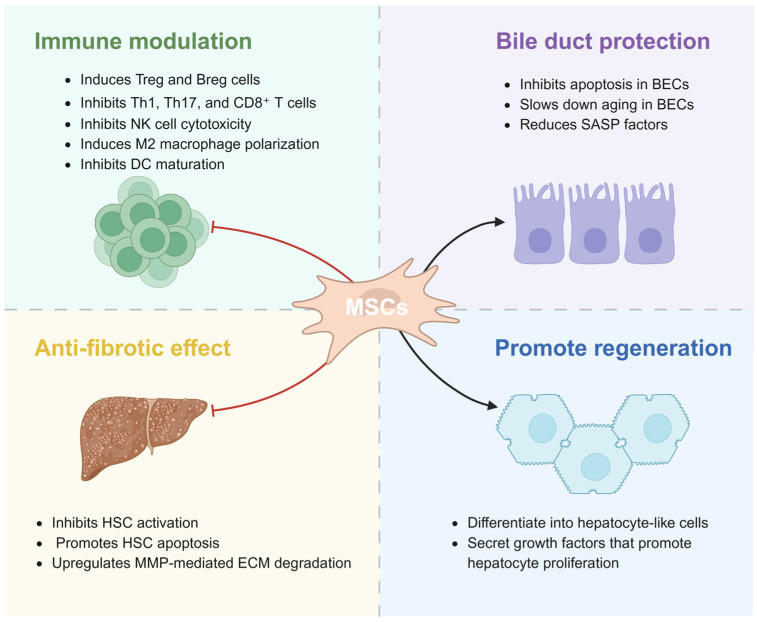
**Multifaceted Therapeutic Benefits of MSCs in PBC.** The four quadrants represent the interconnected benefits of MSCs: immune modulation, bile duct protection, anti-fibrotic effect, and promotion of regeneration. BEC, biliary epithelial cell; Breg, regulatory B cell; DC, dendritic cell; ECM, extracellular matrix; HSC, hepatic stellate cell; MMP, matrix metalloproteinase; MSC, mesenchymal stem cell; NK, natural killer; PBC, primary biliary cholangitis; SASP, senescence-associated secretory phenotype; Th, helper T cell; Treg, regulatory T cell. Created in BioRender. Zhenxia Huang (2026) https://app.biorender.com/illustrations/69c806e44359961d1612b32d?slideId=dabec98a-f244-4441-a8f0-bd472aaafb1c (accessed on 28 March 2026).

In conclusion, MSC-derived EVs and exosomes constitute a cell-free therapeutic strategy for synergistically intervening in the pathological process of PBC through multiple mechanisms, including immune modulation, regeneration promotion, bile duct protection, and anti-fibrotic effect (Table 1). These mechanisms do not act in isolation but are interrelated and mutually reinforcing: immune modulation creates a favorable inflammatory microenvironment for bile duct repair and liver regeneration; anti-fibrotic effects remove physical barriers to tissue regeneration; and the promotion of regeneration fundamentally restores the structural and functional integrity of the liver. To provide a holistic overview of the multifaceted therapeutic benefits of MSCs, we have summarized the key mechanisms (Figure 6). Although challenges remain in clinical translation—including preparation standardization, in-depth mechanism elucidation, and delivery optimization—engineered modifications (e.g., miRNA enrichment or targeted modifications) hold promise for enhancing therapeutic specificity and efficacy. With advancing preclinical and clinical studies, MSC-derived EVs may offer a novel cell-free therapeutic strategy for PBC.

## 4. Current Status and Challenges in Clinical Translation

### 4.1. Current Clinical Trial Evidence

MSC-based therapies have advanced to the clinical trial stage for PBC. To date, three MSC-based clinical trials for PBC have been registered and initiated worldwide (Table 2). Among these, two exploratory studies have been completed and their results published, while a multicenter randomized controlled trial is currently ongoing.

In the two studies with published results, MSC therapy demonstrated a favorable safety profile and preliminary efficacy. Wang et al. treated seven PBC patients with an inadequate response to UDCA using umbilical cord-derived MSCs. Patients received intravenous infusions of 0.5 × 10^6^ cells/kg every four weeks for a total of three doses, followed by 48 weeks of observation. The results showed good tolerability, with only one case of self-limiting fever. By the end of follow-up, serum alkaline phosphatase (ALP) and gamma-glutamyl transferase (GGT) levels were significantly decreased compared to baseline, fatigue and pruritus symptoms showed subjective improvement, and the Mayo risk score remained stable [116]. Subsequently, Wang et al. treated ten UDCA-resistant PBC patients with bone marrow-derived MSCs using a single infusion of 3–5 × 10^5^ cells/kg and followed them for 12 months. No infusion-related adverse reactions were observed, and patients reported significant improvements in pruritus, fatigue, and emotional function. Serum levels of alanine aminotransferase (ALT), aspartate aminotransferase (AST), GGT, and direct bilirubin (DBil) decreased significantly at 3–6 months, and these effects persisted for up to 12 months. Additionally, a reduction in CD8^+^ T cells and an increase in Tregs in peripheral blood were observed, further confirming the immunomodulatory and therapeutic effects of MSCs [117].

Currently, a randomized controlled trial is underway in China, which plans to enroll 140 patients with refractory PBC. The experimental group will receive three MSC infusions (0.1–1 × 10^6^ cells/kg) in combination with UDCA, while the control group will receive a placebo combined with UDCA. The primary endpoint is the change in ALP levels, and secondary endpoints include improvements in other liver function parameters, symptoms, and liver histology.

A comprehensive analysis of existing clinical trials reveals both commonalities and limitations. Both completed studies have confirmed the safety of MSC therapy and suggested its potential efficacy; however, limitations such as small sample sizes, single-arm designs, and the absence of control groups make it difficult to rule out the influence of the disease’s natural course and placebo effects. These limitations also hinder the assessment of individual variations in treatment response. Therefore, conclusions should be interpreted with caution. It is worth noting that in PBC clinical trials, reductions in ALP and GGT have been widely accepted as key biochemical surrogate endpoints for assessing treatment response, and are significantly associated with a reduced risk of liver-related events and prolonged survival [118,119]. Therefore, the improvements in biochemical markers observed in the aforementioned study have potential clinical significance. However, due to the limited follow-up duration and the lack of observation of liver-related endpoints, it is currently unclear whether these improvements will translate into long-term clinical benefit. Current mechanistic studies suggest that the multifaceted regulatory roles of MSCs may underpin their therapeutic potential for PBC. However, translating these mechanisms into definitive clinical benefits requires large-scale randomized controlled trials. Such trials should aim to identify optimal dosing regimens, determine suitable patient subgroups, and incorporate long-term endpoints (such as progression to cirrhosis and liver transplant rates) as primary outcomes.

### 4.2. Challenges in Clinical Translation

#### 4.2.1. Standardization of Cell Source and Preparation

MSCs derived from different tissue sources exhibit significant heterogeneity in immunomodulatory capacity, proliferative potential, and paracrine profiles [120]. More importantly, clinical-grade MSCs cannot be directly used as freshly isolated primary cells and must undergo in vitro expansion and purification to achieve the required quantity and purity. However, the profound impact of culture conditions—such as medium composition, oxygen concentration, and passage number—on cellular function has not yet been fully elucidated, leading to poor comparability of cell products across different studies [121]. Furthermore, there is a lack of unified international standards for MSC quality assessment. Variations in surface marker identification and functional testing methods among different institutions result in inconsistencies in evaluating the quality and efficacy of MSC-based products [122]. Therefore, the establishment of a potency assay-based functional quality control system remains an urgent necessity.

#### 4.2.2. Route of Administration, Optimal Dosage and Treatment Timing

The route of administration significantly influences the biodistribution, therapeutic efficacy, and procedural risks of MSCs. Intravenous infusion is the most commonly used delivery method in current clinical trials due to its simplicity and minimal invasiveness. However, after intravenous injection, the majority of MSCs become entrapped in the pulmonary capillary bed, resulting in poor hepatic homing efficiency [123]. While intrahepatic arterial or portal venous injections could enhance local concentration in the liver, their clinical feasibility remains low. The optimal dosage also remains undetermined; most existing trials have adopted empirical doses of 1–2 × 10^6^ cells/kg, a range borrowed from studies on other autoimmune diseases, lacking support from dose–response studies specific to PBC. Regarding the timing of administration, no studies have yet explored how the disease stage of PBC influences the response to MSC therapy. The maximum benefit of MSCs is typically observed during the early inflammatory phase of the disease rather than the chronic fibrotic stage [124]. Nevertheless, MSC therapy is currently often considered a last resort; ideally, however, it should be initiated before irreversible damage occurs.

#### 4.2.3. Long-Term Safety and Carcinogenic Risk

MSC transplantation carries the risk of adverse side effects. In vitro expansion of MSCs may lead to the accumulation of genetic mutations, and their immunosuppressive effects could potentially weaken the body’s immune surveillance against microscopic tumors [125]. Additionally, allogeneic MSC infusion may induce host immune responses, potentially resulting in diminished therapeutic efficacy or adverse reactions [126]. The lack of long-term follow-up studies leaves the safety assessment incomplete.

#### 4.2.4. Large-Scale Production of MSC-Derivatives

MSC-EV therapy is regarded as a “cell-free” alternative, yet its industrial translation faces production bottlenecks. One of the primary obstacles is the immaturity of isolation and purification technologies—methods such as ultracentrifugation, tangential flow filtration, and size exclusion chromatography each have limitations in yield, purity, and scalability, making it difficult to simultaneously meet the demands for clinical-grade large-scale production [127]. A more fundamental challenge lies in the fact that EVs derived from different sources of MSCs exhibit distinct compositional characteristics. MSCs derived from bone marrow, adipose tissue, umbilical cord, and other sources generate EVs with varying functional potencies—research indicates that their capabilities in angiogenesis, osteogenesis, and immunomodulation differ depending on the tissue of origin [128]. This diversity complicates quality control and leads to inconsistent functional outcomes across different studies. Furthermore, their storage stability and in vivo delivery efficiency require optimization, slowing the clinical translation of MSC-EV therapies compared to initial expectations [129]. It is essential to develop methods for the large-scale production of MSC-EVs that comply with Good Manufacturing Practice standards, enabling the safe transition of cell-free therapies from laboratory research to clinical applications.

#### 4.2.5. Preclinical Models and Their Predictive Validity

A key but unresolved question is whether the mechanisms of MSCs identified in animal models can accurately predict their therapeutic potential in humans. Murine models, such as dnTGFβRII and NOD.c3c4 mice, recapitulate key features including antimitochondrial antibodies and lymphocytic cholangitis. However, they do not fully capture the genetic complexity or immune heterogeneity of primary biliary cholangitis in humans [130]. Moreover, most preclinical studies administer MSCs during the early stages of disease, whereas patients typically receive treatment only after years of asymptomatic progression and often following an inadequate response to UDCA. Whether MSCs retain comparable immunomodulatory efficacy in the presence of established fibrosis, senescent cholangiocytes, or prior pharmacological treatment remains uncertain. Addressing these gaps will require more clinically relevant animal models—such as aged animals or those with established fibrosis—alongside mechanistic studies designed to directly assess whether the effects observed in animals are operational in patients.

## 5. Conclusions and Future Prospects

This review summarizes research progress on the use of MSCs and their derivatives in treating PBC. Results indicate that through multiple mechanisms—including immunomodulation, hepatic differentiation potential, and anti-fibrotic effects—they offer novel therapeutic approaches for PBC patients with poor response to existing drugs. Although positive efficacy has been demonstrated in animal models, most studies remain in the preclinical stage. Given differences between animal models and human disease in immune microenvironments and disease progression, clinical translation of MSCs and their derivatives remains challenging. Key unresolved issues include the isolation, standardized preparation, and quality control of MSC-Exos. To advance clinical application, establishing a comprehensive standardized system—from cell sourcing and culture conditions to exosome extraction and characterization—is essential to enhance research reproducibility and comparability of results. With a deepening understanding of MSC biological functions and Exo mechanisms, subsequent efforts should actively advance standardized clinical trials focusing on evaluating administration routes, therapeutic timing, dose optimization, and long-term safety. In the future, stem cell therapy is expected to further enhance targeting and regulatory precision through engineered modification strategies while ensuring safety and efficacy. These research advances will broaden the application of regenerative medicine in the field of liver diseases.

## Figures and Tables

**Table 1:** Potential Role of Mesenchymal Stem Cell Derivatives in the Treatment of Primary Biliary Cholangitis.

Functional Dimension	Source	Primary Active Component	Functional Description	References
Immune modulation	MSC-EVs	miR-7977	Targeting NF-κB IZ inhibits IκBζ translation, reduces IL-17A production, and thereby suppresses Th17 differentiation	[100]
	MSC-EVs	mitochondrial protein	Metabolic reprogramming of CD4^+^ T cells suppresses glycolysis, enhances oxidative phosphorylation, and reduces their activation	[101]
	WJMSC-sEVs	PD-L1	By binding PD-L1 on the surface of sEV membranes to PD-1 on T cells, it directly inhibits TCR-mediated T cell activation	[108]
	MSC-Exos	miR-223	By stimulating STAT3, it exerts hepatoprotective, anti-inflammatory, and anti-apoptotic effects	[109]
	BMSC-Exos	—	Inhibit B cell and T cell proliferation; regulate B cell mRNA expression; reduce IgM secretion	[98]
	Apoptotic MSC-EVs	—	Inhibits CD3^+^ T cell proliferation, promotes Treg cell differentiation, and drives macrophage polarization toward the M2 phenotype.	[110]
Promote regeneration	BMSC-EVs	miR-146a-5p	Activate small hepatocyte-like progenitor cells within the liver to promote liver regeneration.	[111]
	BMSC-sEVs	miR-20a-5p	Targeting PTEN activates the AKT signaling pathway, reducing hepatocyte apoptosis and promoting their proliferation.	[112]
Bile duct protection	hPMSC-Exos	—	Delaying oxidative stress-induced cholangiocellular senescence and reducing SASP components	[103]
Anti-fibrotic effect	BMSC-Exos	miR-26a, miR-144-3p	Targeting SLC7A11 induces ferroptosis in HSCs and suppresses their activation.	[105,106]
	hUC-MSC-Exos	miR-499a-5p	Targeting ETS1/GPX4-mediaferroptosis in HSCs	[107]
	MSC-Exos	miR-27b-3p	Downregulating YAP inhibits the transcription of its downstream target LOXL2, thereby alleviating fibrosis.	[104]
	ADMSC-EVs	miR-150-5p	Target and suppress CXCL1 expression to attenuate HSCs activation and liver fibrosis.	[113]
	Pd-MSC-EVs	miR-378c	By targeting SKP2 to stabilize E-cadherin, EMT is inhibited, thereby inactivating HSCs.	[114]
	MSC-Exos	circDIDO1	Suppressing HSCs activation by miR-141-3p/PTEN/AKT pathway	[115]

MSC-EVs, Mesenchymal stem cell (MSC)-derived extracellular vesicles (EVs); WJMSC, Wharton’s Jelly-derived MSC; sEVs, small extracellular vesicles; PD-L1, Programmed death-ligand 1; TCR, T cell receptor; Exos, Exosomes; BMSC, Bone marrow-derived mesenchymal stem cell; hPMSC, human placental mesenchymal stem cell; SASP, Senescence-associated secretory phenotype; hUCMSC, human umbilical cord mesenchymal stem cell; HSCs, Hepatocyte stellate cells; ADMSC, Adipose-derived mesenchymal stem cell; Pd-MSC, Pluripotent-derived mesenchymal stem cell; EMT, Epithelial–mesenchymal transition. Note: “—” indicates that the relevant information of this item is not mentioned in the reference.

**Table 2:** Clinical Application of MSC Therapy in PBC.

MSC Source	Intervention (Dose/Route/Frequency/Combination)	Patient Characteristics	Study Number
UCMSCs	Dose: 0.5 × 10^6^ cells/kg Route: Peripheral vein Frequency: 3 times (weeks 0, 4, 8) Combination: Continued UDCA	UDCA treatment for ≥1 year with incomplete response (*n* = 7)	NCT01662973
Allogeneic BMSCs	Dose: 3–5 × 10^5^ cells/kg Route: Intravenous Frequency: Single infusion Combination: Continued UDCA	UDCA treatment for ≥1 year meeting Paris criteria (*n* = 10)	NCT01440309
MSCs	Dose: 0.1–1 × 10^6^ cells/kg Route: Peripheral vein Frequency: 3 times (weeks 0, 4, 8) Combination: Continued UDCA	Refractory PBC (non-responsive to UDCA)	NCT03668145

MSC, Mesenchymal stem cell; PBC, primary biliary cholangitis; UCMSCs, umbilical cord-derived MSCs; BMSCs, bone marrow-derived mesenchymal stem cells; UDCA, ursodeoxycholic acid. Data have been taken from clinicaltrials.gov (last accessed on 22 February 2026).

## Data Availability

No new data were created or analyzed in this study.
